# Intervention Effect of Long-Term Aerobic Training on Anxiety, Depression, and Sleep Quality of Middle School Students With Depression After COVID-19

**DOI:** 10.3389/fpsyt.2021.720833

**Published:** 2021-10-18

**Authors:** Lin Luo, Naiqing Song, Hao Yang, JiaHong Huang, Ling Zhou, Liping Zhang

**Affiliations:** ^1^College of Physical Education, Guizhou Normal University, Guiyang, China; ^2^Basic Education Research Center, Southwest University, Chongqing, China; ^3^East China Normal University-Zhongxu Postdoctoral Workstation, Shanghai, China; ^4^Zhongxu School Affiliated to East China Normal University, Chongqing, China

**Keywords:** aerobic exercise, middle school students, depression, anxiety, sleep quality

## Abstract

**Objective:** To explore the effects of using RPE exercise intensity monitoring methods and 12-week mid- and low-intensity team aerobic training on anxiety, depression and sleep quality of depressed middle school students after the COVID-19 epidemic.

**Methods:** All study participants were all from a boarding middle school in Chongqing, China. All study participants were screened by the self-rating depression scale and reached the diagnostic criteria for depression. The study subjects were divided into a control group (*N* = 35) and an exercise group (*N* = 34). The exercise group performed 30-min night aerobic running four times a week. Use the Borg 6–20 rating of perceived exertion scale (RPE) as a monitoring tool for exercise intensity, and control the exercise intensity at RPE = 11–14. And the control group studied and lived normally. The experiment lasted 12 weeks in total. After the experiment, there were 34 people in the control group and 23 people in the exercise group. The subjects' anxiety, depression and Pittsburgh sleep quality were scored before and after the experiment.

**Results:** After intervention, the depression index (*p* < 0.01) of the exercise group was significantly lower than that of the control group.

**Conclusion:** Using the RPE exercise intensity monitoring method for 12 weeks of mid- and low-intensity team aerobic training can improve the depressive symptoms of depressed middle school students, and it is beneficial to improve the students' mental health.

## Introduction

On December 29, 2019, a new type of coronavirus pneumonia (COVID-19) caused by the SARS-CoV-2 coronavirus was first discovered in Wuhan, China. On 30 January 2020, the WHO Emergency Committee declared a global health emergency based on growing case notification rates at Chinese and international locations. The government of China launched an unprecedented nationwide emergency plan, including the closure of unnecessary businesses, public transportation and schools. In order to reduce the risk of COVID-19 transmission, most middle school students in China had adopted home-based epidemic prevention measures. In the spring semester of 2020, the learning style of most middle school students in China had also shifted from offline learning to online learning. The threat of the spread of COVID-19, the long-term of staying at home, the reduction of going out, the study pressure brought by online learning and the sedentary lifestyle behaviors caused an increase in psychological problems among middle school students (1, 2). Anbao et al. conducted a survey of 2893 middle school students in Anhui, China in February 2020 and found that the incidence of depression among middle school students under investigation was as high as 38.9% (3). With the effective control of the spread of COVID-19 by the Chinese government, most middle schools in China resumed normal offline school life in September 2020. Although the depression of some middle school students has been naturally improved with the relief of the epidemic and the progress of time, some middle school students still have obvious depressive symptoms (4), which could have potentially adverse effects on their study and life.

An increasing number of studies based on meta-analysis show that physical activity (PA) and exercise are useful methods for improving depression symptoms, promoting health outcomes, and preventing other mood disorders (5–8). Laren et al. suggested that exercise may be an important means to improve the emotional health of children and adolescents, and there is almost no difference between high-intensity and low-intensity exercise (9). With the deepening of research, some scholars agree that moderate-to-vigorous aerobic exercise that is performed multiple times a week for 8 weeks or longer can be recommended as the best candidate for ameliorating depression in adolescents (10). However, the effect of low-intensity aerobic exercise on the mood improvement of depressed patients should not be ignored. Meyer et al. reported that 20 min of low-intensity bicycle training can increase the subjective well-being of depressed female subjects (11). For those who do not have enough exercise experience, low or medium intensity aerobic exercise seems easier to develop and persist (12). However, it is worth noting that exercise therapy does not have a significant response effect on all patients with depression. The study of Imboden et al. and Hallgren et al. found that without other treatments for depression, the response rate of depressed patients to active exercise is around 40–50% (13, 14). Although not everyone is sensitive to treatments that improve depression symptoms through exercise, increasing physical activity is still a potentially effective intervention for reducing the risk of depression and relieving depression symptoms.

Although in the past few decades, researchers have conducted a lot of exploration on exercise intervention for depression, reports on various exercise methods and experimental conditions have emerged one after another. However, there are few studies on group exercise intervention for depressed students in an educational environment. Therefore, in order to effectively alleviate the depressive symptoms of middle school students following the COVID-19 epidemic and to improve their quality of life, it is necessary and important to explore a simple, easy, effective, and usable team exercise program for depression intervention for middle school students.

Previous studies have shown that depression is often accompanied by anxiety, and depressed patients often have sleep problems (15). Physical exercise may not only be related to the improvement of individual mental health, regular physical exercise can also improve people's sleep quality (16, 17). In view of this, we attempted to adopt the simple exercise intensity self-monitoring method commonly used by professional athletes in the field of competitive sports to help sports coaches monitor the exercise intensity of depression exercise therapy. Assuming that depressed middle school students use this method to control exercise intensity, 12 weeks of low-to-medium-intensity aerobic running training can significantly improve their depression, anxiety symptoms and sleep status. The purpose of this research is to explore anti-depressive group exercise therapy programs for middle school students in the Chinese educational environment. It is hoped that this group exercise therapy project can improve the psychological problems of depressed middle school students, improve their sleep quality, cultivate exercise habits and help them to develop a healthy lifestyle.

## Methods

### Research Design

This study adopts an experimental design with exercise intervention in the exercise group and no exercise intervention in the control group. The subjects in the exercise group and the control group were all from the same boarding middle school. The exercise group used a low-to-mid-intensity aerobic exercise intervention program lasting 12 weeks, and the control group did not perform this exercise intervention. In addition, the study plan and life plan of the subjects in the exercise group and the control group were the same during school. Subjects in both groups did not receive other antidepressant treatment options. One week before the start of the experiment and 48 h after the final training session, the research observation indicators of the middle school students in the exercise group and the control group were collected. This study was approved by the ethics review of the Academic Committee of the Institute of Physical Education of Guizhou Normal University (No. 20200910).

### Setting and Participants

**Figure 1:**
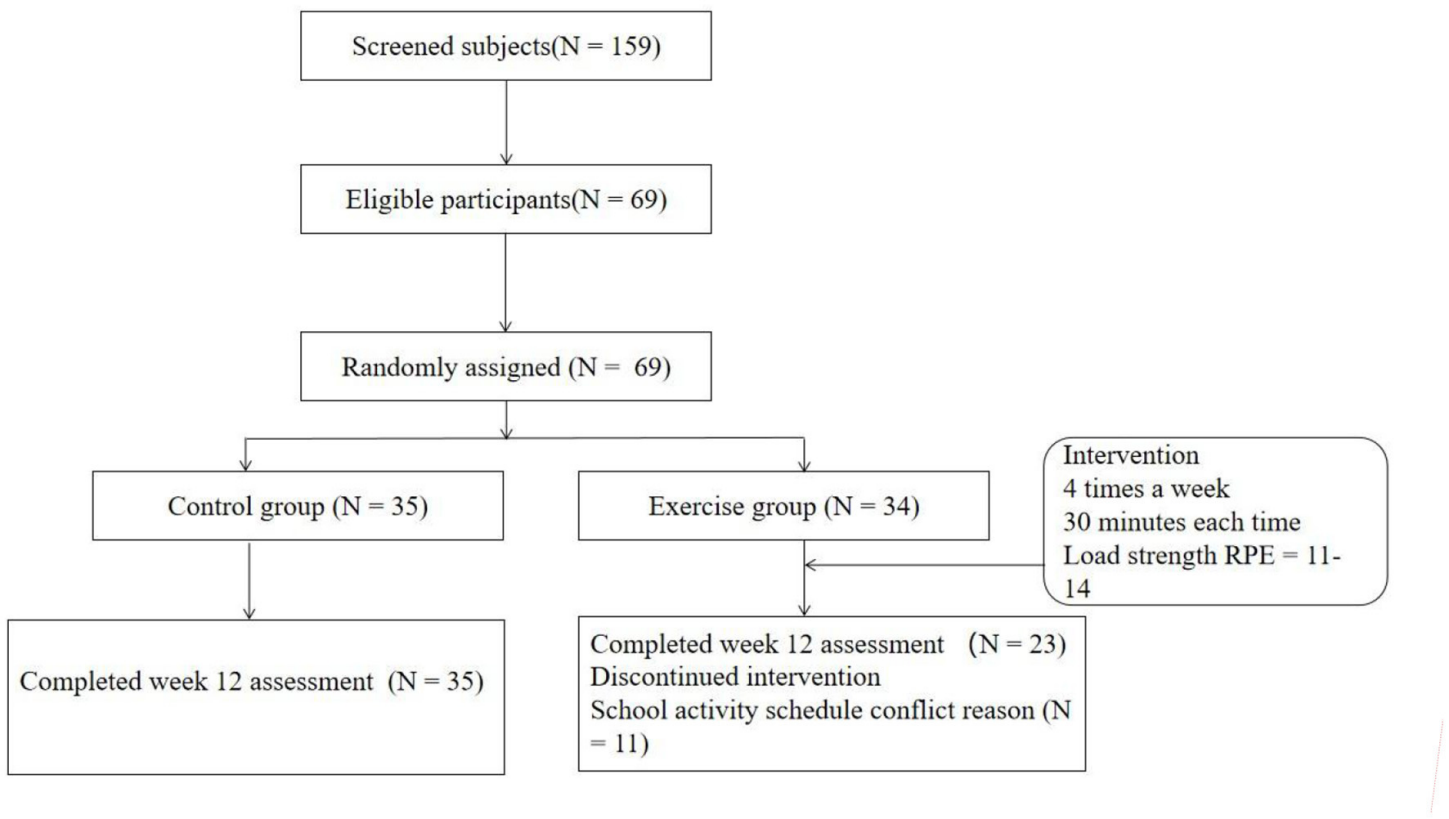
Schematic diagram of aerobic training intervention for 12 weeks.

The research subjects are all middle school students attending a boarding middle school in Chongqing, China. First, after obtaining the informed and verbal consent of middle school students and their legal guardians, 159 middle school students were screened for depression symptoms through the Self-Rating Depression Scale (SDS). A depression index score of 0.50 or higher is the criterion for judging depressive symptoms. A total of 69 middle school students with depressive symptoms were screened through this method. The screened depressed students were randomly divided into exercise group (*N* = 34) and control group (*N* = 35). The students in the exercise group used the physical activity preparation questionnaire (PAR-Q) and cardiovascular disease (CVD) risk factor evaluation methods to assess the exercise risk. The exercise risk of all the students in the exercise group met the research requirements. Students in both groups did not use other treatment options for depression. One week before the start of the experiment, the two groups of students were collected for relevant research indicators. However, 1 week after the experiment started, the 11 students in the exercise group could not guarantee training because the school required to participate in other competition preparation activities. Therefore, these 11 students withdrew from the experiment 1 week after the start of the training. At the end of the 12-week experiment, there were finally 35 people in the control group and 23 people in the exercise group. The difference in pretest depression index data between the 34 initial subjects in the exercise group and the 23 remaining subjects in the exercise group was compared by independent *t*-test, and the difference between the two was not statistically significant. Therefore, in the final statistics, we only counted the data before and after the experiment of the participants in the exercise group who completed the 12-week training. All the exercise interventions were carried out in this school (Figure 1).

### Intervention and Training

All the participants in the exercise group used the Borg 6–20 rating of perceived exertion scale (RPE) (18) to monitor the load of each exercise, and the intensity of each exercise was controlled at RPE = 11–14 (equivalent to low-to-mid intensity). Each training session of aerobic running lasts for 30 min and is carried out for 4 days a week for a total of 12 weeks. The advantage of the RPE as an exercise intensity monitoring method is that not only can it meet the varying intensity of exercise from person to person, but it can also meet the needs of increasing exercise intensity as individual exercise capacity improves. Aerobic running training takes places after the students finish their studies in the evening. Professional sports coaches are responsible for organizing and arranging each exercise training session as well as organizing exercise preparation activities and exercise relaxation training before and after each exercise. In each training session, the school doctor is responsible for the medical supervision during the exercise.

### Instruments and Measurements

This study takes depression as the main outcome of this study, and anxiety and sleep quality as the secondary outcome. RPE is used as an indicator of exercise intensity monitoring.

#### Depression

The self-rating depression scale (SDS) (19), compiled by Professor Zung in 1965, is used to measure the severity of depression. Depression index = cumulative score for each item ÷ 80 (highest original total score). The index ranges from 0.25 to 1.0. The higher the index, the more severe the depression. A depression index score of higher than 0.50 is considered to represent obvious depressive symptoms.

#### Anxiety

The self-rating anxiety scale (SAS) (20), compiled by Professor Zung in 1971, is used to evaluate the subjective feelings of individuals with anxiety symptoms. The original total score multiplied by 1.25 is the standard total score, and the critical value for evaluation is 50 points. The higher the score, the more obvious the anxiety tendency.

#### Sleep Quality

The Pittsburgh sleep quality index (PSQI) (21) was compiled in 1989 by Dr. Buysse, a psychiatrist at the University of Pittsburgh, USA. The total PSQI score uses seven sub-items, namely, subjective sleep quality, time to fall asleep, sleep duration, sleep efficiency, sleep disorders, hypnotic drug use and daytime dysfunction, as indicators for evaluating sleep. Each item is scored on a scale of 0–3, and the cumulative score is the total PSQI score (0–21 points). The higher the PSQI score, the more severe the insomnia.

#### RPE

The rating of perceived exertion (RPE) (18) is a table developed by the Swedish psychologist Brog according to the principles of psychology, and it displays how subjects feel and confirms their load during exercise, which is divided into 6–20 levels. The RPE can be used to check the subjective feeling of fatigue after exercise. In the field of competitive sports, it is also used to monitor the exercise intensity of athletes. In this study, the RPE scale was used to monitor the exercise intensity of the subjects in the exercise group.

The above-mentioned four scale tools have been widely used in China and have been confirmed to have good reliability and validity. SDS, SAS, and PSQI assessments are all carried out by professional psychologists, and RPE assessments are carried out by professional sports coaches. The data of SDS, SAS, and PSQI are collected before and after the intervention (before the test/after the test) to evaluate the changes in mental and sleep aspects to reflect the effectiveness of the program in the mental health and physiological effects of depressed middle school students. RPE data are collected before and after each exercise training session to reflect whether the subjects' exercise intensity meets the requirements of the research design (For specific descriptions of the above four measurement tools, please refer to the Supplementary Materials).

### Analysis

Data analysis was performed using SPSS version 21.0 (IBM Corp., Armonk, NY, USA). The demographic differences between the two groups were analyzed using an independent *t*-test for continuous variable (such as age, height, weight) baseline analysis. The chi-square test was used to analyze the baseline of categorical data (gender, grade). Use a mixed linear model to compare the observational indicators (depression index, anxiety score, PQSI evaluation results) between and pre/post the exercise and control groups. The level of significance was set at *p*< 0.05.

## Results

The main purpose of this study is to explore the effects of using RPE for exercise intensity monitoring and using a mid-to-low intensity 12-week aerobic running team training program on depression, anxiety and sleep in depressed students. According to the research objectives, the following results are presented to prove the validity of the research plan.

### Descriptive Statistics

Table 1 displays the basic information of participants. There was no significant difference in age, gender, height, weight or grade between the two groups of subjects before intervention (*p*> 0.05).

**Table 1:** Participants' basic information.

		**Exercise group**	**Control group**	***X*^2^/*F***	***p*-value**
		**(*N* = 23)**	**(*N* = 34)**		
Gender *n* (%)				0.348	0.555
	Male	52.17%	51.43%		
	Female	47.83%	48.57%		
Age (y)		13.83 ± 0.39	13.96 ± 0.21	−1.421	0.164
Height (cm)		157.78 ± 6.30	157.96 ± 6.81	−0.090	0.929
Weight (kg)		57.97 ± 13.64	56.23 ± 11.89	0.461	0.647
Grade (%)				0.277	0.501
	7	50.94%	48.67%		
	8	49.06%	51.33%		

Table 2 displays the mean and standard deviation of the depression index for both the exercise group and the control group. The data showed that both groups experienced an overall decrease in their depression levels over time. The mean of the depression index at pretest was 0.60 (SD = 0.04) for control group and 0.59 (SD = 0.06) for exercise group. At post-test, the mean of the depression index was 0.56 (SD = 0.07) for control group and 0.44 (SD = 0.13) for exercise group. According to the evaluation rules of SDS, the depression degree of the two groups before the experiment was mild to severe.

**Table 2:** Descriptive statistics of depression at pre- and the post-test for the total sample and by group.

**Samples**	**Pre-test**		**Post-test**	
	$\bar{x}$	**SD**	$\bar{x}$	**SD**
Control group	0.60	0.04	0.56	0.07
Exercise group	0.59	0.06	0.44	0.13

Table 3 displays the mean and standard deviation of the SAS scores for both the exercise group and the control group. The data showed that both groups experienced an overall decrease in their anxiety levels over time. The mean of the SAS scores at pretest was 39.23 (SD = 11.44) for control group and 41.46 (SD = 11.32) for exercise group. At post-test, the mean of the anxiety score was 36.46 (SD = 9.89) for control group and 39.95 (SD = 9.12) for exercise group. According to the evaluation rules of SAS, the anxiety degree of the two groups before the experiment was mild to moderate.

**Table 3:** Descriptive statistics of anxiety at pre- and the post-test for the total sample and by group.

**Samples**	**Pre-test**		**Post-test**	
	$\bar{x}$	**SD**	$\bar{x}$	**SD**
Control group	39.23	11.44	36.46	9.89
Exercise group	41.46	11.32	39.95	9.12

Table 4 displays the means and standard deviations of the measured levels of the Sleep Quality Assessment for both groups. All 7 components (subjective sleep quality, sleep latency, sleep duration, habitual sleep efficiency, sleep disturbances, use of sleep medications, and daytime dysfunction) of the Sleep Quality Assessment as well as the calculated global PSQI were considered. Except sleep efficiency, the other 6 components scores and the global PSQI scores decreased from pretest to post-test for both groups. For sleep efficiency, the scores increased from pretest to post-test for both groups. According to the Sleep Quality Assessment, a global PSQI of 5 or greater is indicative of poor sleep quality. The results showed that both groups had students with poor sleep quality.

**Table 4:** Descriptive statistics of sleep quality at pre- and post-test for the total sample and by group.

**Variables**	**Samples**	**Pre-test**		**Post-test**	
		$\bar{x}$	**SD**	$\bar{x}$	**SD**
Global PSQI	Control group	4.52	2.79	4.13	2.44
	Exercise group	5.52	3.33	3.56	2.71
Subjective sleep quality	Control group	0.60	0.65	0.59	0.32
	Exercise group	1.13	0.81	0.78	0.52
Sleep latency	Control group	0.74	0.81	0.57	0.50
	Exercise group	1.35	0.88	0.78	0.73
Sleep duration	Control group	0.78	0.93	0.65	0.95
	Exercise group	0.43	0.59	0.22	0.51
Sleep efficiency	Control group	1.00	1.16	1.13	1.21
	Exercise group	0.13	0.45	0.34	0.57
Sleep disturbances	Control group	0.52	0.59	0.51	0.50
	Exercise group	0.78	0.79	0.34	0.57
Use of sleep medications	Control group	0.30	0.63	0.00	0.00
	Exercise group	0.00	0.00	0.00	0.00
Daytime dysfunction	Control group	0.69	0.82	0.47	0.73
	Exercise group	1.69	1.02	1.08	1.04

The independent samples *t*-test revealed no significant difference between the exercise group and the control group at pretest for depression (*t* = 0.938, *p*> 0.05), anxiety (*t* = −0.210, *p*> 0.05) and the sleep quality (*t* = −1.106, *p*> 0.05).These results indicated that there was no significant difference in the mean scores of the dependent variables between the exercise group and the control group before the experimental conditioning began.

### 2 × 2 Mixed-Design ANOVA Repeated Measure

As seen in Table 5, the results of the ANOVA revealed a significant main effect of time on the depression test score (*F* = 10.214, *p*< 0.01), indicating that both the exercise group and the control group experienced a significant decrease in their depression index from the pretest to the post-test.

**Table 5:** ANOVA repeated measures of each dependent variable at post-test.

**Variable**		***F*-value**	***p*-value**	$\eta_{\text{p}}^{2}$
Depression	Within group (time)	10.214	0.002	0.193
	Between groups	13.487	0.001	0.235
Anxiety	Within group (time)	0.071	0.792	0.002
	Between groups	2.012	0.163	0.044
Global PSQI	Within group (time)	3.287	0.077	0.070
	Between groups	0.170	0.682	0.004
Subjective sleep quality	Within group (time)	1.561	0.218	0.034
	Between groups	6.162	0.017	0.123
Sleep latency	Within group (time)	5.425	0.025	0.110
	Between groups	7.273	0.010	0.142
Sleep duration	Within group (time)	0.063	0.803	0.001
	Between groups	6.907	0.012	0.136
Sleep efficiency	Within group (time)	0.676	0.415	0.015
	Between groups	23.672	0.000	0.350
Sleep disturbances	Within group (time)	2.239	0.142	0.048
	Between groups	0.028	0.868	0.001
Use of sleep medications	Within group (time)	5.284	0.026	0.107
	Between groups	5.284	0.026	0.107
Daytime dysfunction	Within group (time)	5.124	0.029	0.104
	Between groups	16.530	0.000	0.273

The ANOVA results also presented a significant difference in the depression index by group (*F* = 13.487, *p*< 0.01), meaning that experiment intervention mattered in the decrease of depression score. The mean total score for the exercise group decreased significantly more than the mean total score for the control group.

The results demonstrated no significant difference of time on the anxiety test score (*F* = 0.071, *p*> 0.05).Similarly, the results presented no significant difference in anxiety scores by group (*F* = 2.012, *p*> 0.05).The results showed that the 12-week mid- and low-intensity aerobic training intervention used in this study had no significant effect on alleviating students' anxiety.

The ANOVA results showed no significant difference of time on the global PSQI test (*F* = 3.287, *p*> 0.05). The results revealed no significant difference between the global PSQI test score by group (*F* = 0.170, *p*> 0.05). Some components showed much more significant changes than others. Subjective sleep quality score display no significant change of time (*F* = 1.561, *p*> 0.05).There were significant differences in the subjective sleep quality between the exercise group and the control group before the experiment (*F* = 6.162, *p*< 0.05).The results revealed a significant change over time (*F* = 5.425, *p*< 0.05) or by group (*F* = 7.273, *p*< 0.05) on sleep latency score. The mean score for the exercise group decreased significantly less than the mean score for the control group. Sleep duration score display no significant change of time (*F* = 0.063, *p*> 0.05).There were significant differences in the sleep duration between the exercise group and the control group before the experiment (*F* = 6.907, *p*< 0.05).Sleep efficiency score display no significant change of time (*F* = 0.676, *p*> 0.05).There were significant differences in the sleep efficiency between the exercise group and the control group before the experiment (*F* = 23.672, *p*< 0.05).Sleep disturbances did not display a significant change over time (*F* = 2.239, *p*< 0.05) or by group (*F* = 0.028, *p*< 0.05). The results revealed a significant main effect of time on the use of sleep medications score (*F* = 5.284, *p*< 0.01), indicating that both the exercise group and the control group experienced a significant decrease in the score of use of sleep medications from the pretest to the post-test. Daytime dysfunction displayed a significant change over time (*F* = 5.124, *p*< 0.05) or by group (*F* = 16.530, *p*< 0.05).

## Discussion

Physical activities and exercises designed to relieve depression and anxiety and improve sleep include aerobic exercise, resistance exercise, Tai Chi and meditation (22, 23). Although many studies have provided convincing evidence that exercise has a certain positive effect as an improvement of depression, the research on the dose effect of exercise beneficial to depression needs to be further expanded. In fact, the most difficult problem in developing exercise intervention programs for depressed patients in a real environment may be the problem of monitoring exercise intensity. If the exercise intensity is too small, it may be difficult to produce significant stimulation to the body, and it will not produce beneficial physical and mental effects. Excessive exercise intensity may cause participants to give up because of difficulty in execution. Chinese scholar Lou Hu's meta-analysis of 62 studies on the treatment of depression with different exercise methods found that small/medium-intensity exercise has the highest effect value, and the effect values of medium-intensity exercise and high-intensity exercise are similar, reaching the intermediate clinical effect level (23). The heart rate intensity of the small/medium-intensity aerobic exercise recommended by the American Academy of Sports Medicine for most people is about 30–60% HRR (24). However, without real-time heart rate monitoring equipment, this intensity is considerably difficult to monitor. The RPE method used in this study to monitor exercise intensity is a subjective feeling based on heart rate intensity and has a very wide range of applications in the field of competitive sports (25). The authors of the current paper attempted to introduce it into this study to provide assistance in monitoring the intensity of the subjects throughout the exercise cycle. To date, it has been very successful. The results of this study prove that the 12-week mid-to-low-intensity aerobic team running training using RPE monitoring is a safe, effective, and simple exercise treatment program that can help depressed middle school students improve their depressive symptoms.

After 12 weeks of aerobic training, the depression index of the exercise group was lower than that of the control group, indicating that the low-to-medium-intensity aerobic running exercise program used in this study can help to alleviate depression in middle school students. These results show that the intervention plan is effective in improving the mental health of depressed middle school students. Although after 12 weeks of aerobic training, there was no statistically significant difference in anxiety levels and overall PSQI scores between the exercise group and the control group, the scores of most components of sleep quality in the exercise group still showed a downward trend. It shows that the team aerobic exercise intervention program used in this study still has a certain effect on improving the sleep quality of middle school students with depression. Although the results of this study on anxiety and sleep quality are inconsistent with the conclusions of some existing studies (26), it was speculated that this may be related to the test schedule after the experiment, which is just before the students' final academic assessment. Before facing academic assessment, students may be more prone to anxiety and poor sleep.

According to the data before and after the experiment in the exercise group, after 12 weeks of aerobic running, the students in the exercise group had an improved depression index. The indicators significantly improved, indicating that long-term aerobic training with medium and low intensity had a positive effect on relieving depression in middle school students in the exercise group and on improving sleep quality. Although the differences in the depression index, and PSQI score in the control group before and after the experiment were not statistically significant, it can be observed that these indicators also improved after the experiment. This may be related to the relief of many stressors of depressed middle school students in the control group after COVID-19 was effectively controlled. However, it also shows that although the psychological problems and sleep problems of depressed middle school students were alleviated to a certain extent with the reduction of stress stimulation, this kind of self-relief is slow and its effect is limited. It is worth noting that before the experiment, there were significant differences in multiple dimensions of PSQI between the exercise group and the control group, which also shows that the performance of depression with sleep problem have obvious individual differences.

This research still encounters various limitations. For example, when we assess the sleep quality of middle school students, we only use the PSQI self-assessment results as the basis for judgment, instead of using some objective sleep assessment tools. At the same time, no further analysis of the elimination of mental disorders or sleep disorders in middle school students has been carried out. This may partially affect our research conclusions. Another limitation is the timing of the experiment, the practice environment and practice projects, and the sample size of the research. When we started our research, Chinese middle schools had just returned to normal offline learning soon. At that time, it was very difficult to obtain permission to conduct team sports intervention research in the educational environment. And our research samples are all urban middle school students. Whether middle school students in rural areas will have the same psychological problems and intervention effects, this research still cannot answer these questions. Nevertheless, we still hope that when the research conditions permit, we can try our best to maintain a better quality control of the research process and advance the research. In order to obtain a feasible plan for team sports to intervene in depression. Of course, in the future, more explorations can be made on the intervention of team sports in adolescent depression, such as more diversified sports options, which may help students stimulate more interest in exercise and help form good exercise habits.

## Conclusions

In summary, although the secondary outcomes (anxiety, sleep quality) observed in this study did not show significant differences between the two groups before and after the experiment, the primary result (depression) was significantly reduced. This showed that by using the RPE exercise intensity monitoring method, the 12-week low-to-mid intensity team aerobic training for depressed middle school students can improve their depressive symptoms. Therefore, it is possible to combine the spare time of middle school students with some simple exercise methods and the promotion of exercise knowledge to encourage those with depression symptoms to improve their physical exercise behaviors, which may result in the relief of psychological and sleep troubles caused by depression. This study identified a healthy coping strategy that depressed middle school students can apply to their daily lives to help reduce depression. This research provides design ideas and experience for the design of group exercise programs for depressed students in an educational environment.

## Data Availability Statement

The raw data supporting the conclusions of this article will be made available by the authors, without undue reservation.

## Ethics Statement

The studies involving human participants were reviewed and approved by the Academic Committee of the Institute of Physical Education of Guizhou Normal University (No.20200910). Written informed consent to participate in this study was provided by the participants' legal guardian/next of kin.

## Author Contributions

LL conceived the research, designed research tools, participated in data collection, performed data analysis, and interpretation. LL and NS drafted the manuscript. HY participated in data collection and experimental training. JH participated in the data analysis. LZho and LZha participated in the documentation. All authors have read and approved the final manuscript.

## Funding

This research was strongly supported by Zhongxu School Affiliated to East China Normal University. The funding for this research came from the Guizhou Provincial Philosophy and Social Science Project Fund (No. 19GZYB121), the East China Normal University-Zhongxu Postdoctoral Workstation Fund (No. 2019001), and the Guizhou Provincial Department of Education Youth Growth Project Fund (Qianjiao He KY [2021] 291) Funding.

## Conflict of Interest

The authors declare that the research was conducted in the absence of any commercial or financial relationships that could be construed as a potential conflict of interest.

## Publisher's Note

All claims expressed in this article are solely those of the authors and do not necessarily represent those of their affiliated organizations, or those of the publisher, the editors and the reviewers. Any product that may be evaluated in this article, or claim that may be made by its manufacturer, is not guaranteed or endorsed by the publisher.
