# Supplementary material for: Intervention Effect of Long-Term Aerobic Training on Anxiety, Depression, and Sleep Quality of Middle School Students With Depression After COVID-19
Source: Front Psychiatry. 2021 Oct 18;12:720833. doi: 10.3389/fpsyt.2021.720833 (PMC8558258; doi:10.3389/fpsyt.2021.720833)
Supplement: Supplementary file 1 [file Table_1.DOC]

**Supplementary file 1：**

| **Pittsburgh Sleep Quality Index (PSQI) Table** | | | | | |
| --- | --- | --- | --- | --- | --- |
| **No.** | **Item** | **Score** | | | |
|  |  | 0 point | 1 point | 2 point | 3 point |
| 1 | In the past 1 month, go to bed at night usually at _____ o'clock | | | | |
| 2 | In the past month, it usually takes ______min from going to bed to falling asleep | □≤15min | □16~30min | □31~60min | □≥60min |
| 3 | In the past 1 month, usually get up at _____ in the morning | | | | |
| 4 | In the past 1 month, I usually sleep _____h every night (not equal to bedtime) | | | | |
| 5 | In the past 1 month, I was troubled by the following conditions affecting sleep | | | | |
|  | a.Difficulty falling asleep (cannot fall asleep within 30 minutes) | □None | □＜11 time/week | □1~2 times/week | □≥3 times/week |
|  | b. Waking up easily or early at night | □None | □＜1 time/week | □1~2 times/week | □≥3 times/week |
|  | c. Go to the toilet at night | □None | □＜1 time/week | □1~2 times/week | □≥3 times/week |
|  | d. Poor breathing | □None | □＜1 time/week | □1~2 times/week | □≥3 times/week |
|  | e. High cough or snoring | □None | □＜1 time/week | □1~2 times/week | □≥3 times/week |
|  | f. Feeling cold | □None | □＜1 time/week | □1~2 times/week | □≥3 times/week |
|  | g. Feeling hot | □None | □＜1 time/week | □1~2 times/week | □≥3 times/week |
|  | h. Having nightmares | □None | □＜1 time/week | □1~2 times/week | □≥3 times/week |
|  | i. Pain and discomfort | □None | □＜1 time/week | □1~2 times/week | □≥3 times/week |
|  | j. Other things that affect sleep | □None | □＜1 time/week | □1~2 times/week | □≥3 times/week |
|  | If yes, please specify: | | | | |
| 6 | In the past 1 month, in general, what do you think of your sleep quality: | □Well | □Better | □Poor | □Very bad |
| 7 | In the past month, how did you use medication to hypnotize: | □None | □＜1 time/week | □1~2 times/week | □≥3 times/week |
| 8 | In the past month, have you often felt sleepy? | □None | □＜1 time/week | □1~2 times/week | □≥3 times/week |
| 9 | Have you insufficient energy to do things in the past month? | □Rarely | □Occasionally | □Sometimes | □Often |

**Supplementary file 2：**

Please read each item carefully to understand the meaning, and then according to your actual feelings in the last week, choose the answer that suits you best and tick the corresponding number with "√".

1. No or very little time 2. A small part of the time 3. A considerable amount of time 4. Most or all of the time)

| **The self-rating depression scale（SDS）** | | | | |
| --- | --- | --- | --- | --- |
| 1. I feel unhappy and depressed | 1□ | 2□ | 3□ | 4□ |
| 2. I think the morning of the day is the best | 1□ | 2□ | 3□ | 4□ |
| 3. I cry or feel like crying | 1□ | 2□ | 3□ | 4□ |
| 4. I don't sleep well at night | 1□ | 2□ | 3□ | 4□ |
| 5. I eat as much as usual | 1□ | 2□ | 3□ | 4□ |
| 6. I feel as happy as ever when I have close contact with the opposite sex | 1□ | 2□ | 3□ | 4□ |
| 7. I noticed that my weight has dropped | 1□ | 2□ | 3□ | 4□ |
| 8. I am suffering from constipation | 1□ | 2□ | 3□ | 4□ |
| 9. My heartbeat is faster than usual | 1□ | 2□ | 3□ | 4□ |
| 10. I feel tired for no reason | 1□ | 2□ | 3□ | 4□ |
| 11. My mind is as clear as usual | 1□ | 2□ | 3□ | 4□ |
| 12. I don’t think it’s difficult to do things often | 1□ | 2□ | 3□ | 4□ |
| 13. I feel upset and unable to calm down | 1□ | 2□ | 3□ | 4□ |
| 14. I have hope for the future | 1□ | 2□ | 3□ | 4□ |
| 15. I get angry more easily than usual | 1□ | 2□ | 3□ | 4□ |
| 16. I think it is easy to make a decision | 1□ | 2□ | 3□ | 4□ |
| 17. I think I am a useful person, someone needs me | 1□ | 2□ | 3□ | 4□ |
| 18. My life is very interesting | 1□ | 2□ | 3□ | 4□ |
| 19. I think others will live better if I die | 1□ | 2□ | 3□ | 4□ |
| 20. I am still interested in things that I usually are interested in. | 1□ | 2□ | 3□ | 4□ |

**Supplementary file 3：**

Please read each item carefully to understand the meaning, and then according to your actual feelings in the last week, choose the answer that suits you best and tick the corresponding number with "√".

（1.No or very little time 2. A small part of the time 3. A considerable amount of time 4. Most or all of the time)

| **The self-rating anxiety scale (SAS)** | | | | |
| --- | --- | --- | --- | --- |
| 1.I feel more nervous and anxious than usual | 1□ | 2□ | 3□ | 4□ |
| 2. I feel scared for no reason  3. I get upset or frightened easily | 1□ | 2□ | 3□ | 4□ |
| 4. I think I might be going crazy | 1□ | 2□ | 3□ | 4□ |
| 5. I think everything is fine and no misfortune will happen | 1□ | 2□ | 3□ | 4□ |
| 6. My hands and feet tremble | 1□ | 2□ | 3□ | 4□ |
| 7. I am troubled by headache, neck pain and back pain | 1□ | 2□ | 3□ | 4□ |
| 8. I feel weak and tired easily | 1□ | 2□ | 3□ | 4□ |
| 9. I feel calm and easy to sit quietly | 1□ | 2□ | 3□ | 4□ |
| 10. I feel my heart beating very fast | 1□ | 2□ | 3□ | 4□ |
| 11. I am distressed because of dizziness | 1□ | 2□ | 3□ | 4□ |
| 12. I have a fainting episode, or feel like fainting | 1□ | 2□ | 3□ | 4□ |
| 13. I feel very easy to breathe in and exhale | 1□ | 2□ | 3□ | 4□ |
| 14. My hands and feet are numb and tingling | 1□ | 2□ | 3□ | 4□ |
| 15. I am troubled by stomach pain and indigestion | 1□ | 2□ | 3□ | 4□ |
| 16. I often have to urinate | 1□ | 2□ | 3□ | 4□ |
| 17. My hands and feet are often dry and warm | 1□ | 2□ | 3□ | 4□ |
| 18. My blush is hot | 1□ | 2□ | 3□ | 4□ |
| 19. I fall asleep easily and sleep well all night | 1□ | 2□ | 3□ | 4□ |
| 20. I have nightmares | 1□ | 2□ | 3□ | 4□ |

**Supplementary file 4：**

| **The rating of perceived exertion (RPE)** | | |
| --- | --- | --- |
| RPE | Subjective sensory characteristics | Relative Strength（%） |
| 6 |  | 0.0 |
| 7 | Very,very,light | 7.1 |
| 8 |  | 14.3 |
| 9 | Very light | 21.4 |
| 10 |  | 28.6 |
| 11 | Fairly light | 35.7 |
| 12 |  | 42.9 |
| 13 | Somewhat hard | 50.0 |
| 14 |  | 57.2 |
| 15 | Hard | 64.3 |
| 16 |  | 71.5 |
| 17 | Very hard | 78.6 |
| 18 |  | 85.8 |
| 19 | Very,very,hard | 95.0 |
| 20 |  | 100.0 |
